# Prevalence and cutaneous comorbidities of hidradenitis suppurativa in the German working population

**DOI:** 10.1007/s00403-020-02065-2

**Published:** 2020-04-22

**Authors:** Natalia Kirsten, Nicole Zander, Matthias Augustin

**Affiliations:** Institute for Health Services Research in Dermatology and Nursing (IVDP), University Medical Center Hamburg-Eppendorf (UKE), Martinistraße 52, 20246 Hamburg, Germany

**Keywords:** Atopy, Psoriasis, Epidemiology, Comorbidity, Health care

## Abstract

The association of hidradenitis suppurativa with other skin diseases has not yet been investigated in larger studies based on dermatological exams. The objectives of this study are to determine the prevalence and cutaneous comorbidities of hidradenitis suppurativa in the German working population. Between 2014 and 2017, 20,112 people in 343 German companies were examined for the presence of clinical features of hidradenitis suppurativa within the framework of a cross-sectional epidemiological study based on whole-body examinations. In addition, all cutaneous comorbidities were recorded. Point prevalence was calculated and the differences between individuals with and without hidradenitis suppurativa were determined by bivariate analysis. All statistical procedures were performed using SPSS 23.0 for Windows. Of 20,112 people examined, mean age was 43.6 ± 10.5 years; 52.3% were male. In total, *n* = 57 people (0.3%) with hidradenitis suppurativa were identified; 61.4% (*n* = 35) being male. In addition, non-inflammatory hidradenitis suppurativa-related lesions were found in 674 other individuals. In a bivariate comparison, patients with hidradenitis suppurativa showed significantly more frequently the following cutaneous comorbidities: acne vulgaris, psoriasis, seborrhoeic dermatitis, excoriations, and folliculitis. We determined a point prevalence of hidradenitis suppurativa of 0.3%. Since we have examined the working population, the healthy worker effect, which could have led to underestimation of prevalence, cannot be ruled out. The point prevalence of 0.3% for employed people in Germany and a prevalence of 3.0% for inflammatory and non-inflammatory hidradenitis suppurativa-related lesions show that hidradenitis suppurativa is an important disease for the whole health system.

## Introduction

Hidradenitis suppurativa (HS) is a chronic inflammatory skin disease that has become the focus of scientific interest in recent years. Although first described by Velpeau in 1839 [24], it was not until 2015 that the first European S1 guideline for the treatment of HS was published [26]. According to the Dessau definition (1st International Conference on Hidradenitis Suppurativa, March 30–April 1, 2006, Dessau, Germany), the disease is defined by recurrent inflammatory nodules, abscesses, and fistulas which occur chronically in typical localisations. The predilection sites for inflammatory lesions are, in most cases, the body folds and body sites that are subject to increased friction, such as the inner thighs.

HS causes substantial disease burden to the affected people. Severe pain, movement restrictions, and scarring, which are partly caused by large-scale surgery, but also secretions and bad odor, emotional and psychosocial impairment lead to severe restrictions in health-related quality of life [14, 16]. In routine health care, marked delays in finding the right diagnosis are common. The literature reports a diagnostic delay of up to 7.2 ± 8.7 years [19].

The prevalence data vary between 0.053% and 4% depending on the studies [4, 8, 17, 22]. The studies differ in methods and target population. Most publications derive from secondary data analyses such as claims data or from surveys based only on self-assessments of the interviewees. Thus, the true prevalence of HS remains unclear.

HS is associated with numerous comorbidities. In addition to polycystic ovary syndrome [6], chronic inflammatory bowel diseases [3], spondylarthritis [18], or depression [13]. The metabolic syndrome [15] also plays an important role in patients with HS. In some cases, HS occurs as part of a symptom complex in combination with other diseases such as in PASH (pyoderma gangrenosum, acne, and suppurative hidradenitis) or PAPASH (pyogenic arthritis, pyoderma gangrenosum, acne, and suppurative hidradenitis) [7, 21]. Even if the pathogenesis of HS has not yet been definitively clarified, the association with other inflammatory diseases points to the immunological genesis of the disease in addition to the response to anti-inflammatory therapy.

Associations with the other skin diseases were mainly investigated for syndromatic forms of HS. An association with acne vulgaris in PASH or polycystic ovary syndrome is well described. The same is true for the pyoderma gangrenosum. In a study by Kridin et al. [9], a significantly higher prevalence of HS was found in people with psoriasis compared to people without psoriasis.

The association of HS with other skin diseases has not yet been investigated in larger studies, in particular not in primary data derived from dermatological exams.

For this reason, the aim of this study was to determine the prevalence of HS in the German working population and to investigate the association of HS with other skin diseases.

## Patients and methods

### Study design and people examined

This cross-sectional epidemiological study is based on whole-body examinations of employees in German companies between 2014 and 2017. In total, 20,112 people were examined in 343 German companies from different branches of industry.

All clinical investigations were conducted according to the principles expressed in the Declaration of Helsinki. According to the Good Practice of Secondary Data Analysis, no approval of an ethical committee is required.

### Assessments

The skin screenings were voluntary and free for the employees. The clinical exams were performed at working hours by trained dermatologists. Besides of a short history and the evaluation of general dermatological findings, the people were checked for a defined list of HS-related lesions. The lesions were divided into those indicating the current inflammatory activity of HS and postoperative or postinflammatory scarring.

Inflammatory lesions included: single abscesses, confluent abscesses, inflammatory nodules, fistulas, inflammatory papules, and open comedones.

The diagnosis of HS was considered most probable when one of the above inflammatory lesions was applied.

In addition, other pathological skin findings as well as the skin type after Fitzpatrick were recorded. The findings were recorded in a standardized electronic record by a medical assistant.

### Statistics

All statistical procedures were performed using SPSS 23.0 (IBM, Armonk, New York) for Windows. Descriptive analyses of the cohort characteristics including non-adjusted point prevalences were performed, followed by multivariate analyses. All variables were treated as dichotomous variables. The missing values were evaluated as not present. Statistical differences between the participants with and without HS lesions were determined using a bivariate analysis (Chi-square test). A logistic regression for the associated comorbidity was not possible due to the small number of cases in the HS group.

## Results

### Demographics

Overall, 20,112 people were examined between 2014 and 2017. Mean age was 43.6 ± 10.5 years, 52.3% were male (Table 1). In total, *n* = 57 people (0.3%) with HS were identified, 61.4% (*n* = 35) of them being male. There were no age-related differences between age-groups 16–34 years and 35–64 years.

**Table 1:** Socio-demographic and clinical characteristics of working people with and without hidradenitis suppurativa (*N* = 20,112)

	Male		Female		Total	
	*n*	%	*n*	%	*N*	%
Participants	10,517	52.3	9595	47.7	20,112	100.0
Age, years (mean ± SD)	45 ± 10.3		42.1 ± 10.5		43.6 ± 10.5	
Hidradenitis suppurativa	35	0.3	22	0.2	57	0.3

Categorized by phototype, the highest prevalence of HS (0.4%) was found in people with phototype IV (*n* = 515). The lowest prevalence (0.2%) was observed in patients with phototype I (*n* = 1034). However, the groups were too small to allow statements on significance. Phototypes V and VI were represented with 37 people. Among them were no people with HS lesions.

### Prevalence of lesions indicative of HS

**Fig. 1:**
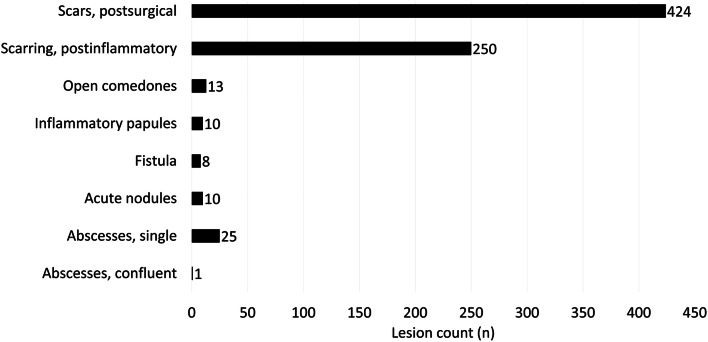
Count of dermatological findings indicative of hidradenitis suppurativa in the German working population (*N* = 20,112, including *n* = 57 with hidradenitis suppurativa)

A total of 612 (3.0%) individuals had at least one inflammatory or non-inflammatory lesion (Fig. 1). Of the inflammatory lesions, abscesses (*n* = 25) were the most frequent, followed by open comedones (*n* = 13), inflammatory papules (*n* = 10) and acute nodules (*n* = 10), fistulas (*n* = 8), and confluent abscess (*n* = 1). In addition, non-inflammatory 674 HS-related lesions were found in 555 other individuals. Postoperative scars were more frequent (*n* = 424) than scarring after inflammation (*n* = 250).

### Cutaneous comorbidities associated with HS

In a bivariate comparison, patients with HS showed significantly more frequently the following cutaneous comorbidities (Table 2): acne vulgaris, psoriasis, seborrhoeic dermatitis, excoriations, and folliculitis. The largest difference between patients with and without HS was found for folliculitis (people with HS 26.3%; people without HS 9.0%).

**Table 2:** Cutaneous comorbidities of hidradenitis suppurativa (HS), *N* = 20,112

Comorbidities	Prevalence				*p* (χ^2^**)**
	People without HS (*n* = 20,055)		People with HS (*n* = 57)		
	*n*	%	*n*	%	
Acne vulgaris	503	2.51	7	12.28	0.000
Atopic dermatitis	360	1.80	1	1.75	0.982
Dermatitis of intertrigines	85	0.42	0	0.00	0.622
Exsiccation eczema	618	3.08	1	1.75	0.562
Hand eczema	129	0.64	1	1.75	0.296
Contact dermatitis	22	0.11	0	0.00	0.802
Psoriasis	421	2.10	4	7.02	0.010
Rosacea	409	2.04	0	0.00	0.276
Seborrhoeic dermatitis	657	3.28	8	14.04	0.000
Excoriations	159	0.79	2	3.51	0.022
Folliculitis	1796	8.96	15	26.32	0.000
Pyoderma	10	0.05	0	0.00	0.866

## Discussion

The aim of our study was to determine the prevalence of HS in the working population in Germany. To the best of our knowledge, this is the first large-scale cross-sectional study of that kind based on physical examination. Overall, we determined a point prevalence of HS of 0.3%. This is largely in accordance with previously published data by Shalom et al. [20]. However, it should be noted that our data represent a point prevalence and most publications are 1-year prevalences derived from the secondary data analysis [19].

Including the scarring associated with HS, we even found the presence of HS-related lesions in 3.0% of individuals. HS is a chronic inflammatory disease that occurs in relapses. The frequency of relapses varies from patient to patient. Primary lesions such as inflammatory nodules or minor abscesses often leave no scars during spontaneous remission. If the abscesses are split or if major surgical interventions are carried out or if inflammation persists for a longer period of time, scarring occurs in the regions of the body area affected by inflammation. Therefore, on one hand, we have looked very closely for current inflammatory lesions to determine the point prevalence of HS in our cohort. However, this does not take into account all patients with HS who currently have no inflammatory relapse. On the other hand, we have also currently considered non-inflammatory lesions associated with HS to estimate possible lifetime prevalence. Since we have included individuals of different ages in our cohort, it can be assumed that some of the individuals may develop HS later and that the patient who had lesions prior to the treatment but healed without scarring was not considered. This means that the overall lifetime prevalence of HS is expected to be significantly higher.

In contrast to general findings, men in the cohort which we examined were more frequently affected by HS than women. However, it should be noted that, as mentioned above, most data on prevalence were obtained from the analysis of secondary data and, therefore, possible gender-specific differences, such as willingness to present oneself to a doctor, were not taken into account. Interestingly, a few smaller studies from the Asian region also showed an increased prevalence of HS in men [10, 12, 25].

Our data show that HS, previously considered a rare disease, should become more important in medical care to minimize burdens for those affected. However, our work also has a few limitations. We have investigated the working population, so data on the unemployed population are missing. Patients with active severe form of HS are often not able to work. Calao et al. could show that persons with HS were significantly more likely to be unemployed [2]. Theut Riis et al. were able to show that persons with HS had received cash benefits or sick pay more often than persons without HS [22]. The same working group identified in their survey-based study an unemployment rate of 25.1% in the HS population versus 5.9% in the general population [23]. Overall, persons with HS show a lower socioeconomic status [1, 5, 11]. This means that a healthy worker effect could have occurred and with this an underestimation of prevalence of HS.

Another limitation is the voluntary character of the study. People rejecting skin examinations might potentially show altered frequencies of skin affections. HS is associated with a severe reduction in quality of life and social isolation. It is possible that especially people with a severe HS did not dare to participate in the screenings.

Even if we do not have data on the localisation of the individual lesions, it can be assumed that data from trained dermatologists are to be evaluated as valid. In addition to the prevalence analysis, we found an association of HS with inflammatory dermatoses such as acne vulgaris, psoriasis, seborrhoeic dermatitis, or folliculitis. A logistic regression analysis was not performed due to the small number of cases in the HS group. Nonetheless, the identified comorbidity profile supports the immunological genesis of the HS disease. Our data are also supported by the work of other research groups, such as Kridin et al. [9], whose working group could show an association of psoriasis with HS. The association of HS with acne vulgaris, especially in the context of syndromes such as PASH [9], PAPASH [21], or in the presence of polycystic ovary syndrome [3], has also been described more frequently. The very frequent presence of folliculitis may indicate that it is a symptom of the disease in HS patients or an early manifestation of HS.

Although our data cannot be directly transferred to the general population, the large cohort of 20,112 people makes it reasonable to assume that they are representative for the German working population.

The point prevalence of 0.3% for employed people in Germany and a prevalence of 3.0% for inflammatory and non-inflammatory HS-related lesions shows that HS is an important disease for the entire health care system. The large diagnostic delay of up to seven years additionally emphasizes the importance of education and need of awareness to intervene in the early phases of the disease. Thus, late consequences such as movement restrictions or scarring of whole-body areas could be avoided and the quality of life of the affected people could be improved, and costs to the payers and the patients be saved.
